# Gut microbiome changes due to sleep disruption in older and younger individuals: a case for sarcopenia?

**DOI:** 10.1093/sleep/zsac239

**Published:** 2022-10-02

**Authors:** Jordi Morwani-Mangnani, Panagiotis Giannos, Clara Belzer, Marian Beekman, P Eline Slagboom, Konstantinos Prokopidis

**Affiliations:** Section of Molecular Epidemiology, Department of Biomedical Data Sciences, Leiden University Medical Center, Leiden, The Netherlands; Department of Life Sciences, Faculty of Natural Sciences, Imperial College London, London, UK; Laboratory of Microbiology, Wageningen University, Wageningen, The Netherlands; Section of Molecular Epidemiology, Department of Biomedical Data Sciences, Leiden University Medical Center, Leiden, The Netherlands; Section of Molecular Epidemiology, Department of Biomedical Data Sciences, Leiden University Medical Center, Leiden, The Netherlands; Department of Musculoskeletal Biology, Institute of Life Course and Medical Sciences, University of Liverpool, Liverpool, UK

**Keywords:** gut microbiome, bacterial diversity, aging, sleep, sarcopenia

## Abstract

Major hallmarks of functional loss, loss of metabolic and musculoskeletal health and (multi)morbidity with aging are associated with sleep disturbances. With poor sleep shifts in gut microbial composition commonly manifest, which could mediate the pro-inflammatory state between sleep disturbances and sarcopenia. This systematic review presents the recent evidence on how sleep disturbances throughout the lifespan associate with and contribute to gut microbial composition changes, proposing a mechanism to understand the etiology of sarcopenia through sleep disturbances. The relationship between disturbed sleep and clinically relevant gut microbiota composition on health aspects of aging is discussed. A search was performed in PubMed, Cochrane Library, Scopus, Web of Science using keywords including (microbio* OR microflora) AND (sleep OR sleep disorder). Six cross-sectional population-based studies and five experimental clinical trials investigating healthy individuals with ages ranging from 4 to 71 were included. The cross-sectional studies reported similarities in associations with sleep disturbance and gut microbial diversity. In older adults, shorter sleep duration is associated with an increase in pro-inflammatory bacteria whereas increasing sleep quality is positively associated with an increase of beneficial Verrucomicrobia and Lentisphaerae phyla. In young adults, the effect of sleep disruption on gut microbiome composition, specifically the ratio of beneficial Firmicutes over Bacteroidetes phyla, remains contradictory and unclear. The findings of this review warrant further research in the modulation of the gut microbiome linking poor sleep with muscle-catabolic consequences throughout the lifespan.

## Introduction

Aging is a biological process encompassing the accumulation of cellular-level damage [1]. Older adults ≥ 65 years old experience a progressive deterioration of musculoskeletal function, that may, in part, be explained by exacerbated sleep disorders followed by metabolic and public health repercussions [2, 3]. Particularly, age-related sleep changes that could lead to sleep fragmentation and overall lower sleep efficiency, may cause metabolic alterations favoring myostatin, cortisol, and insulin resistance, inducing muscle protein catabolism [4]. These changes may be precursors in driving negative metabolic health effects in musculoskeletal physiology including sarcopenia [5]. Globally, an increasingly aged population has been presently observed, employing an urgent awareness of the health impact sleep disturbances may pose on older populations [6].

Accruing sleep architecture changes have been demonstrated throughout the lifespan [7]. In an effort to understand the complexity of sleep through indicators for quality, efficiency, and duration, self-report questionnaires [i.e. Pittsburgh Sleep Quality Index (PSQI)] have been developed [8]. During aging, sleeping patterns are characterized by the decline of all sleep parameters, especially sleep efficiency. For instance, older adults have exhibited decreased slow-wave sleep (deep sleep), early awakening, and fragmented sleep [9, 10]. Evidence in young adults already alights to the importance of sleep in preserving muscle mass. One night of total sleep deprivation is sufficient to promote anabolic resistance and favor muscle catabolism by blunting muscle protein synthesis [11]. Sleep disturbance is conducive to muscle atrophy via a reduction in plasma testosterone and increase in cortisol as replicated in animal models [12]. Hence, the increased prevalence of sleep disorders in older populations may in part account for the aggravating age-related metabolic ramifications.

Recent evidence has proposed a prominent role of gut microbiome alterations associated with changes in sleep architecture and subsequent sleeping disorders [13–15]. The gut microbiome consists of a range of bacteria, viruses, archaea, and fungi. Most data available describes gut bacteria as one of many factors that maintain physiological homeostasis through the production of short-chain fatty acids (SCFA) [16]. Bacterial diversity affects the relative production of different SCFAs and is thus a key factor in understanding gut health. The general consensus is that a diverse bacterial profile is essential in maintaining a healthy physiology, where less diverse microbiomes are associated with gut dysbiosis and different metabolic conditions [17]. Certain profiles of bacteria may have more beneficial or deleterious effects on health [18, 19]. A compositional profile with an abundance of pro-inflammatory bacteria may lead to systemic low-grade inflammation, ultimately leading to activation of skeletal muscle-catabolic pathways observed in sarcopenia [20, 21].

Until now, the majority of the evidence has investigated the relationship of gut bacteria and sleep architecture. However, this association is poorly understood in terms of how it changes throughout the lifespan and how it could impact musculoskeletal degeneration through aging. In this systematic review, we address how sleep parameters including duration, quality, and efficiency associate with the gut microbiota composition throughout the lifespan and extrapolate possible implications on musculoskeletal dysfunction manifesting in sarcopenia. We utilize data from observational studies to examine the association and experimental clinical trials to investigate the directionality of the association.

## Methods

This systematic review was conducted according to the Preferred Reporting Items for Systematic Reviews and Meta-Analyses (PRISMA) guidelines [22]. The protocol was registered in the International Prospective Register of Systematic Reviews (PROSPERO) (Registration number: CRD42022308654).

### Search strategy and screening

Two independent researchers examined peer-reviewed literature published in PubMed, Cochrane Library, Scopus, and Web of Science from January 2000 to January 2022, using MeSH terms that combined any of the following: “microbiota”, “microflora”, “intestinal flora”, “gut dysbiosis”, “fecal microbiota”, “sleep”, and “sleep disorder”. The full search strategy is described in detail in Supplementary Materials (Table S1). The authors screened the titles and abstracts of the articles. If bacterial composition was reported in taxonomic terms, the full-texts were screened according to the eligibility criteria.

### Study eligibility

Articles included in this systematic review had to: (1) be observational or experimental clinical studies, (2) have analyzed gut microbiome composition, (3) recruited healthy participants and/or those with sleep disorders, and (4) have collected data on sleep parameters including sleep duration, efficiency and/or quality. Studies were excluded if: (1) participants had chronic comorbidities.

### Quality assessment

Two authors assessed the methodological quality of the studies using three separate tools for cross-sectional population-based studies, randomized controlled clinical trials (RCTs) and non-randomized controlled clinical trials (NRCTs). These checklists all appraise the validity, results, and generalizability of the studies. The tools thoroughly examined the impact of confounders in the quality of results and conclusions.

The binary AXIS checklist was used to assess the quality of cross-sectional studies, consisting of 20 questions divided into (1) Introduction, (2) Methods, (3) Results, (4) Discussion, and (5) Other [23].

The risk of bias in RCTs was assessed utilizing the Cochrane risk of bias (RoB 2) tool. Risk of bias appraisal included the assessment of bias domains such as: (1) randomization process, (2) deviations from intended interventions, (3) missing outcome data, (4) measurement of the outcome, and (5) selection of the reported result [24]. According to the scoring system, study quality was defined as low risk of bias, some concerns, or high risk of bias.

The Cochrane Risk Of Bias In Non-Randomized Studies—of Interventions (ROBINS-I) tool was used to evaluate NRCTs according to the following domains: (1) bias due to confounding, (2) bias in selection of participants, (3) bias in classification of intervention, (4) deviations from intended intervention, (5) bias due to missing data, (6) bias in measurement outcomes, (7) bias in selection of the reported results [25].

## Results

### Search results

**Figure 1.**
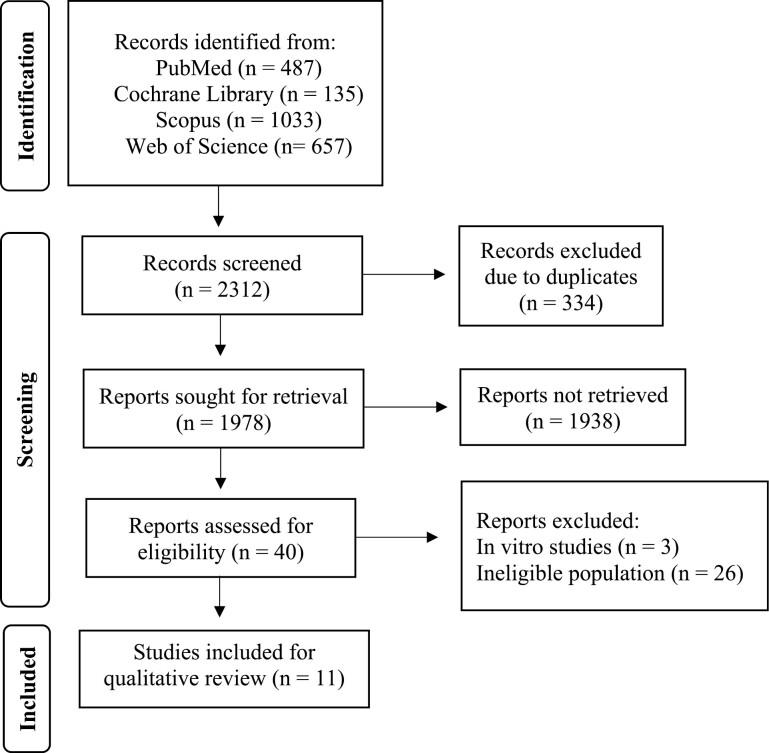
Flow diagram of the included searches of databases and registers.

Literature search produced a total of 2312 articles. After removing 336 due to duplicates, 1978 reports were sought for retrieval, from which 40 full-texts were reviewed. Of the 40 articles, 29 were excluded, from which, three were in vitro studies. Moreover, 26 articles studied populations with chronic comorbidities with or without sleep disorders and deemed ineligible. Overall, 11 studies met the inclusion criteria for this systematic review (Figure 1).

### Study characteristics

Data were extracted to detail the study characteristics such as publication year and type of study. Further documented methodological characteristics included participant characteristics (sample size and age); methods (in terms of measurements for gut microbiome profiling and sleep parameters); study design (branches), primary outcomes; results; and overarching conclusion. Tables for both observational and clinical studies can be found in Tables 1 and 2, respectively. Special attention was given to the age of the studied populations including children (0–6 years), young adults (18–25), middle-aged adults (26–54) and older adults (55–75). Sex differences may occur however the samples sizes of the studies presented negligible differences.

**Table 1.** Study and participant characteristics of the included observational studies

Study year	Study design	Total	Sleep-disturbed		Comparator					
		*n* (M/F)	*n* (M/F)	Age (SD)	*n* (M/F)	Age (SD)	Assessment method	Study duration	Outcomes	Reported results
Wang 2022	Cross-sectional	68 (32/36)	68 (32/36)	4.4 (0.5)	-	-	16s rRNA Actigraphic monitoring	3 days	GM composition Sleep efficiency	Low vs. High TST, SE, WASO: = Alpha diversity Low vs. High TST: – *Bifidobacterium* – *Parabacteroides* – *Turicibacter* + *Blautia* + *Lachnospiraceae* Low vs. High SE: – *Ruminiclostridium* – *Bacteroides* – *Eubacterium ruminantium* + *Coprococcus 1* Low vs. High WASO: = Beta diversity + *Bacteroides* + *Eubacterium ruminantium* – *Coriobacteriales Incertae Sedis*
Agrawal 2021	Cross-sectional	63 (60/3)	16 (15/1)	59.4 (7.5)	47 (45/2)	62.7 (5.8)	16s rRNA sequencing Sleep questionnaires	12 months	GM composition Sleep duration	Short vs. Normal sleepers: = Alpha diversity – Beta diversity – *Firmicutes* = *Bacteroidota* – *Acidaminococcaceae* – *Rikenellaceae* – *Sutterellaceae* – *Rhodospirillales* – *Desulfovibrionaceae* + *Pseudomonadaceae* + *Pasteurellaceae*
Fei 2021	Cross-sectional	652 (251/401)	154 (79/75)	35.6 (6.2)	498 (326/172)	34.7 (6.4)	16s rRNA sequencing Sleep questionnaires	36 months	GM composition Sleep duration	Short vs. Normal sleepers: – Alpha diversity = Beta diversity + Butyrate synthesis + *Dialister* + *Bacteroides*
Grosicki 2020	Cross-sectional	28 (17/11)	9 (6/3)	28.8 (10.0)	19 (11/8)	30.3 (10.8)	16s rRNA sequencing PSQI	1 month	GM composition Sleep quality	PSQI inversely associated with diversity PSQI was positively associated with: +*Firmicutes/Bacteroidetes* ratio + *Blautia* + *Ruminococcus* – *Prevotella*
Smith 2019	Cross-sectional	26 (26/0)	26 (26/0)	22.2 (3.1)	-	-	16s rRNA sequencing Actigraphic monitoring	1 month		Sleep efficiency and duration were positively correlated with: Gut microbiota richness and diversity + *Bacteroidetes* + *Firmicutes* WASO was negative correlated with: – Gut microbiota richness and diversity – *Bacteroidetes* – *Blautia* – *Lachnospiraceae* – *Oribacterium*
Anderson 2017	Cross-sectional	37 (10/27)	37 (10/27)	64.6 (7.5)	-	-	16s rRNA sequencing PSQI	1 month	GM composition Sleep quality	PSQI was positively associated with: +*Verrucomicrobia* +*Lentisphaerae*

GM, gut microbiota; PSQI, Pittsburgh Sleep Quality Index; SE, sleep efficiency; TST, total nighttime sleep; WASO, Wakefulness After Sleep Onset. + indicates increased; - indicates decrease; = indicates no change

**Table 2.** Study and participant characteristics of the included experimental studies

Study year	Study design	Total	Sleep-disturbed		Comparator					
		*n* (M/F)	*n* (M/F)	Age (SD)	*n* (M/F)	Age (SD)	Assessment method	Study duration	Outcomes	Reported results
Wang 2021	RCT, Crossover	25 (13/12)	25 (13/12)	22.2 (0.3)	25 (13/12)	22.2 (0.3)	16s rRNA sequencing Actigraphic monitoring	1 month	GM composition Sleep duration	Sleep deprivation vs. Baseline: – Alpha diversity – Beta diversity – *Prevotella* – *Sutterella* – *Parasutterella* – *Alloprevotella* – *Anaeroplasma* – *Elusimicrobium* – *Allobaculum*
Liu 2020	Clinical Trial	22 (14/8)	22 (14/8)	25.3 (4.5)	-	-	16s rRNA sequencing Sleep-wake cycle shift	11 days	GM composition	Sleep distruption vs. Baseline: *+ Firmicutes/Bacteroidetes* ratio *+ Fusobacteria* *+ Tenericutes* *+ Mollicutes* – *Odoribacter* – *Pasteurellales* – *Clostridiales* *= Bacteroides* *= Parabacteriodes*
Reutrakul 2020	RCT, Crossover	8 (1/7)	8 (1/7)	32.4 (4.6)	8 (1/7)	32.4 (4.6)	16s rRNA sequencing PSQI ESS	4 weeks	GM composition Sleep duration	Low sleep vs. Sleep extension: *=* Alpha diversity *=* Beta diversity – *Tenericutes*
Zhang 2017	Clinical Trial	11 (6/5)	11 (6/5)	37.6 (8.8)	-	-	16s rRNA sequencing Sleep questionnaire	22 days	GM composition Sleep quality	Short vs. Normal sleep: *=* Gut microbiota richness *+ Firmicutes/Bacteroidetes* ratio *+ Fusobacteria* *+ Proteobacteria*
Benedict 2016	RCT, Crossover	9 (9/0)	9 (9/0)	23.3 (0.6)	9 (9/0)	23.3 (0.6)	16s rRNA sequencing Embla A10 recorders	5 days	GM composition Sleep duration	Short vs. Normal sleep: – *Tenericutes* *+ Coriobacteriaceae* *+ Erysipelotrichaceae* *= Firmicutes* *= Actinobacteria* *= Bacteroidetes* *= Euryarchaeota* *= Verrucomicrobia* *= Proteobacteria* *= Cyanobacteria* *= Lachnospiraceae* *= Ruminococcaceae* *= Bifidobacteriaceae* *= Streptococcaceae* *= Prevotellaceae* *= Bacteroidaceae*

ESS, Epworth Sleepiness Scale; GM, gut microbiota; PSQI, Pittsburg Sleep Quality Index. + indicates increased; - indicates decrease; = indicates no change

### Quality assessment of the included studies

Results from a critical appraisal of the methodological quality of eligible observational studies are presented in Table 3. All six cross-sectional studies presented robust study designs. Within the realm of methodology, all studies had a thorough statistical reasoning and reproducibility. However, only one of the six studies justified how the sample sizes were derived through appropriate power calculations [26]. None of the studies provided measures used to address any non-responders—participants who did not provide sufficient data. Moreover, all studies presented adequately described and consistent results. The majority of studies had no conflicts of interest aside for one study [27] that was conducted in African-origin adults. It is important that the inherent cross-sectional design poses an issue of temporality, rendering the association between sleep indicators and gut microbiome composition non-directional and speculative.

**Table 3.** Quality assessment of the six included cross-sectional studies according to the AXIS tool

Study	Q1	Q2	Q3	Q4	Q5	Q6	Q7	Q8	Q9	Q10	Q11	Q12	Q13	Q14	Q15	Q16	Q17	Q18	Q19	Q20
Smith 2019	X	X		X	X	X	X		X	X	X	X				X	X	X		
Grosicki 2020	X	X		X	X	X	X		X	X	X	X	X			X	X	X		
Anderson 2017	X	X	X	X	X	X	X		X	X	X	X	X			X	X	X		
Agarwal 2021	X	X		X	X				X	X	X	X	X			X	X	X		
Fei 2021	X	X		X	X	X	X		X	X	X	X	X			X	X	X	X	X
Wang 2022	X	X		X	X	X	X		X	X	X	X	X			X	X	X		X

Q1: Clear aims; Q2: Appropriate study design; Q3: Justified sample size; Q4: Defined targeted population; Q5: Appropriate population sample; Q6: Representative participant selection; Q7: Non-responders categorization measures; Q8: Measured outcome variables related to aims; Q9: Measured outcome variables with previously trialed measurements; Q10: Clear tools to determine statistical significance; Q11: Described reproducible methods; Q12: Adequately described basic data: Q13; Concerns of response rates for non-responders: Q14; Described information about non-responders: Q15: Internally consistent results; Q16: Presented results for all analyses in methods; Q17: Justified conclusions with results; Q18: Discussed limitations; Q19: Concerns about conflict of interest; Q20: Attained ethical approval or participant consent.

**Figure 2.**
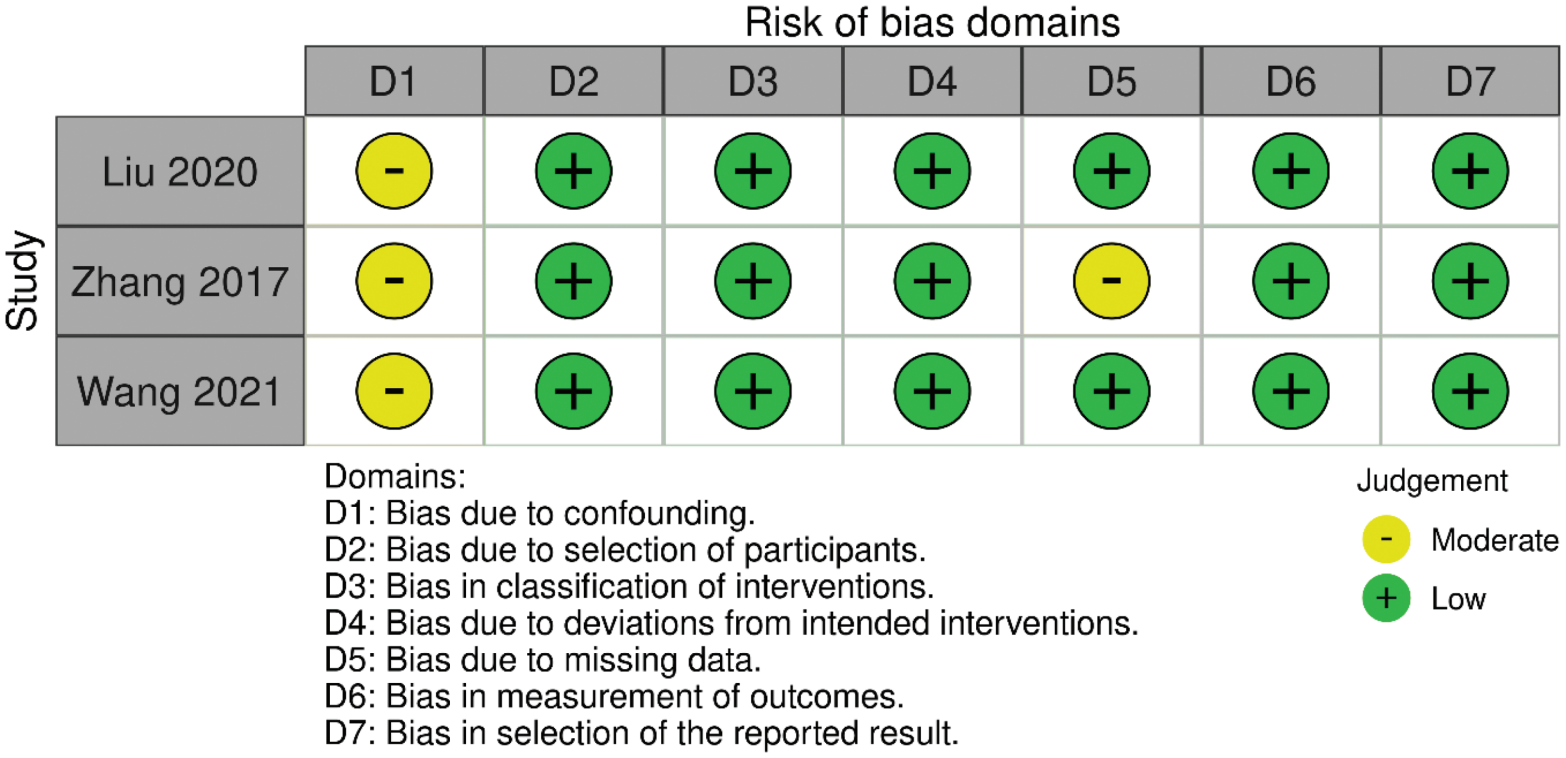
Quality assessment of the three included NRCT studies according to the Cochrane ROBINS-I tool.

The critical appraisal of the three NRCTs using the ROBINS-I tool presented low to moderate risk of bias (shown in Figure 2). All three studies were subjected to bias due to their exploratory nature as the topic of sleep disturbance on gut microbiome composition has not been studied in depth. For this reason, neither study attempted to control any confounding factors such as age or sex that could have influenced the results. While the study from Wang et al. [13]. was fairly homogeneous in terms of age, sex, and body mass index (BMI), participants were not assessed for history of probiotic and antibiotic use. Furthermore, one study [28] had an additional risk of moderate bias due to missing data, in which data from three participants were excluded from analyses due to absence of fecal sample delivery at given time-points. There were no sensitivity analyses performed to reveal any potential changes due to the missing data.

**Figure 3.**
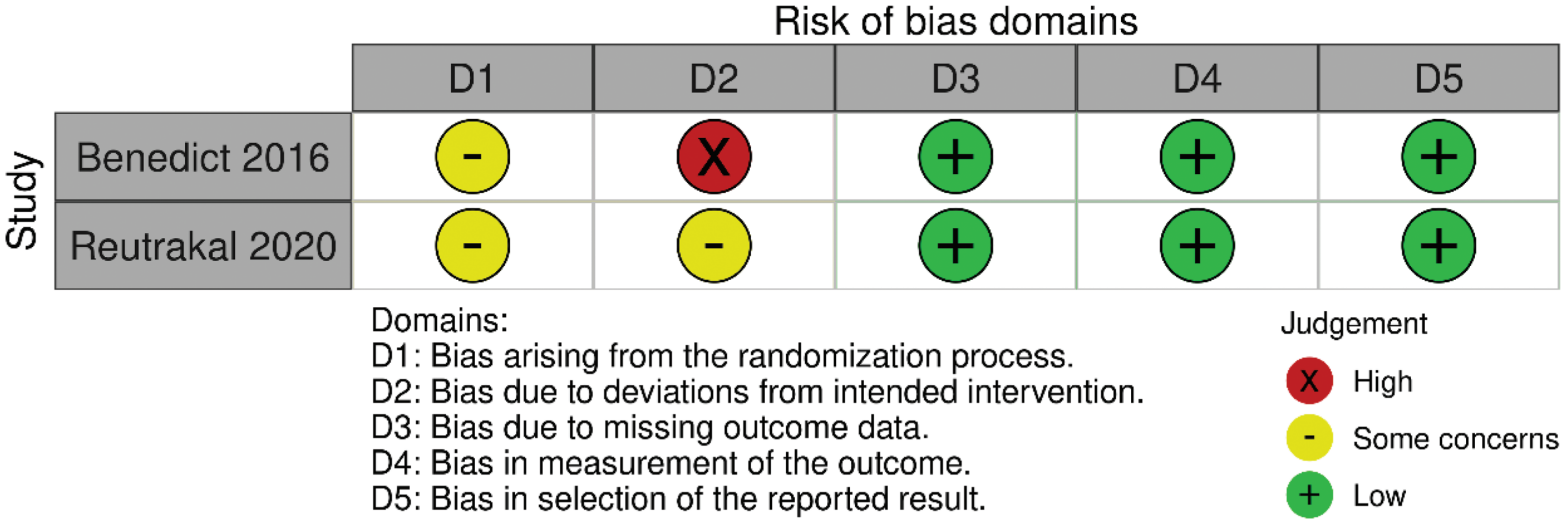
Quality assessment of the one included RCT studies according to the Cochrane RoB 2 tool.

**Figure 4.**
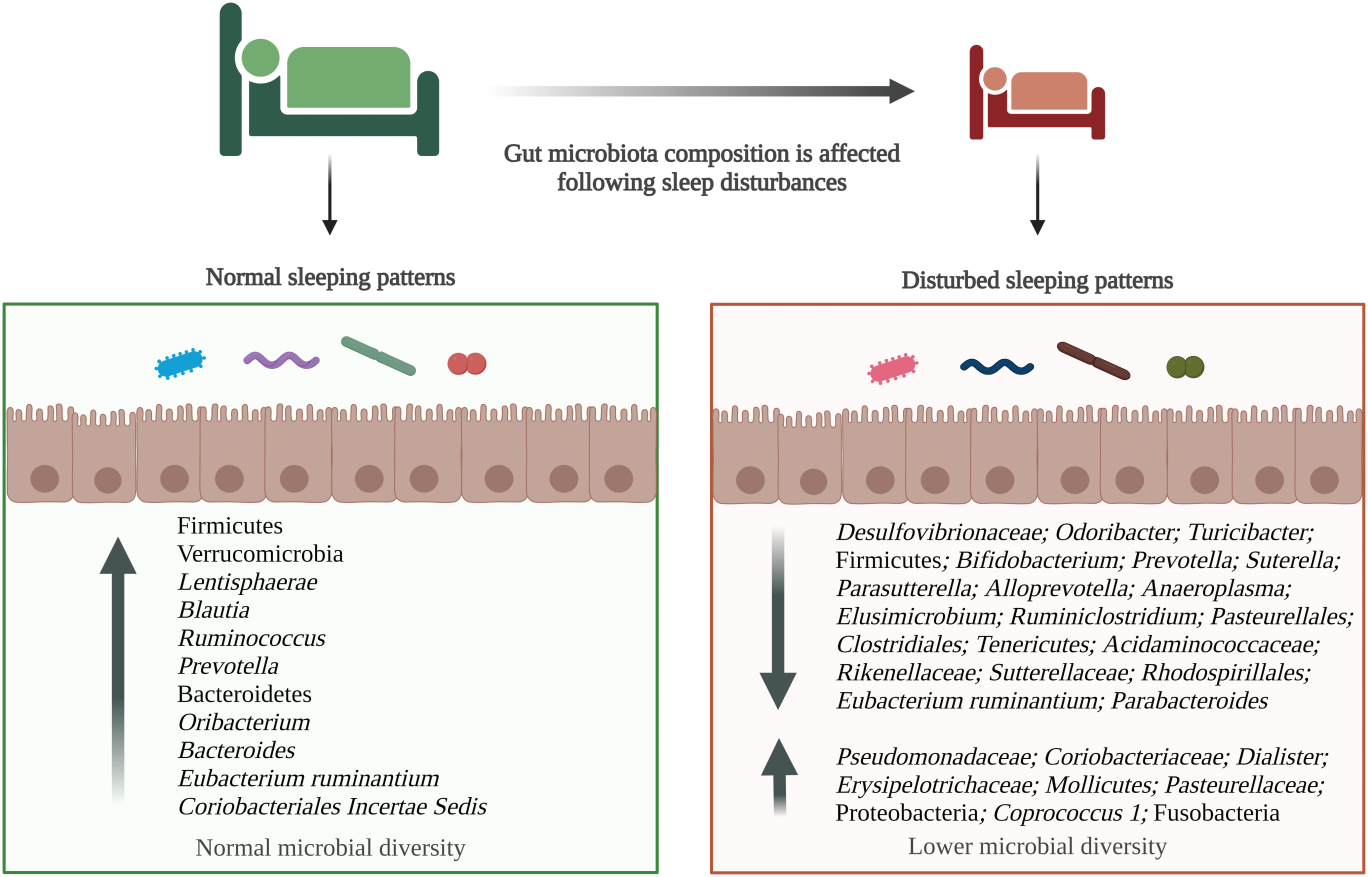
Potential microbial changes in healthy individuals following sleep disturbance.

The two cross-over RCTs [29, 30] were evaluated using the RoB2 tool adapted to cross-over trials, which revealed a certain degree of concern for bias (Figure 3) [24]. There were some concerns regarding the randomization of the allocation sequence as both studies were executed within another ongoing study (Figure 4). Moreover, there were only nine subjects in Benedict et al.’s [29] study, six of which started in the sleep-deprived group and the remaining three in the normal sleep condition group. The period effects emerging from this imbalance were not addressed during analyses. Reutrakul et al.’s [30] research was a secondary data analysis on eight individuals who met their criteria from the original 21 participants in the cross-over study. However, no information regarding the balance of randomization and allocation was disclosed, thus elevating some concerns for the study.

### Results from observational studies

The six observational cross-sectional population-based studies described study outcomes in terms of diversity and taxa abundance for gut bacteria and duration, quality, and efficiency for sleep parameters.

### Bacterial diversity × sleep quality

Two studies reported contradicting results in terms of bacterial diversity and sleep quality. One actigraphy-based study investigating a young adult population noted an association between lower sleep quality in terms of wake after sleep onset and lower bacterial diversity [15]. However, a study also conducted in young adults found an inverse relationship between sleep quality using a PSQI questionnaire and bacterial diversity [31].

### Bacterial diversity × sleep efficiency and duration

Only one actigraphy-based study in young adults determined that a higher bacterial diversity was associated with higher sleep efficiency and duration. Sleep efficiency was specifically positively correlated with bacterial richness and bacterial diversity in the Bacteroidetes phylum and only richness in the Firmicutes phylum [15].

### Taxa abundance × sleep quality

Two studies reported specific changes in bacterial taxa abundances correlating with sleep quality in younger and older adults. In one study, younger individuals reporting superior sleep quality had a higher relative abundance of the Firmicutes phylum, namely the groups *Ruminococcus* and *Blautia*. In contrast, these individuals also had a lower relative abundance of the Bacteroidetes phylum, particularly the group *Prevotella* [31]. In another study with healthy older adults, individuals reporting superior sleep quality had a higher relative abundance of the Verrucomicrobia and Lentisphaerae phyla [26].

### Taxa abundance × sleep efficiency

Only two studies correlated taxa abundance with sleep efficiency in young adults and children. Most notably bacteria belonging to the genus *Lachnospiraceae*, *Corynebacterium*, and *Blautia* were negatively correlated with sleep efficiency in young adults [15]. Another study identified a higher *Bacteroides* relative abundance with sleep efficiency in children [32].

### Taxa abundance × sleep duration

Three studies investigated the effect of sleep duration on taxa abundances. The first study [32] investigated the role of sleep using actigraphy in association with gut microbiome composition in preschool aged children. High sleep duration was associated to higher relative abundance of *Bifidobacterium* genus. Moreover, shorter sleep duration was related to lower relative abundances of *Blautia* genus.

Another cross-sectional study further investigated the effect of sleep duration on gut microbiome composition in middle-aged adults [27]. Similar to the above study, participants were clustered into three categories including short sleepers (<7 h per night), normal sleepers (7 to <9 h per night), and long sleepers (*>*9 h per night). The *Dialister* genus within the Firmicutes phylum was both detected at higher relative abundances in both short and long sleepers. Long sleepers additionally had a superior abundance of Firmicutes bacteria belonging to the *Erysipelotrichaceae*, *Ruminococcaceae*, *Oscillospira*, and *Catenibacterium* families.

A third study [33] investigated the association of sleep duration with gut microbiome composition in older adults, in which participants were clustered into either short (<6 h of sleep per night) or normal sleepers (6–8 h of sleep per night). The results highlighted a significant change in abundance of bacteria under the Proteobacteria phylum. Specifically, a lower abundance of *Suturella* in normal sleepers (1.25%) compared to short sleepers (0.38%) was displayed. In contrast, an increased *Pseudeomonas* abundance was found in normal sleepers (0.08%) as opposed to short sleepers (0.14%).

### Results from experimental clinical studies

Evidence from Liu et al. [34]. suggests that altering the sleep-wake cycles in younger people is associated to shifting gut microbiome profiles. The study collected and analyzed fecal samples at three stages: baseline (7 days of normal sleep), disturbance (one night postponing sleep by 2–4 h), and recovery (2 nights of normal sleep). Results showed that sleeping disturbance led to modest changes in gut microbiome composition. There was no significant baseline variation between the participants, where the increase in specific bacteria during the intervention was then restored similar to baseline levels. Therefore, overnight sleep disturbance is not sufficient to produce significant gut microbiome composition alterations. There was a noticeable increase in phyla Fusobacteria and Tenericutes, and classes Fusobacteria and Mollicutes. The *Odoribacter* and *Bacetoroides* genus classes were identified as the prime drivers of microbial shifts by influencing the abundance of other bacteria at the genus level.

Zhang et al. [28]. conducted an extensive two-round sleep restriction protocol and assessed its impact on gut microbiomec omposition in healthy adults. The protocol involved one round of 5 nights of 4 h sleep and one round of 5 nights with 12 h sleep, followed by a second round of 5 days of 4 h sleep, with a final night of 12 h sleep. Results did not reveal any significant gut bacterial diversity shifts due to sleep deprivation. Specific analyses on all bacterial taxonomic levels did not show any significant changes composition or relative abundances throughout the two rounds of sleep restriction. Further stratification analyses in males did not provide any significant differences.

Benedict et al. [29]. examined the shift in gut composition in nine participants undergoing two nights of sleep deprivation followed by two nights of recovery. The results demonstrated an increased abundance of bacteria within the Firmicutes phylum, in addition to increases in *Coriobacteriaceae* and *Erysipelotrichaea* families, and a decrease in *Tenericutes* in a sleep-deprived state. No significant baseline variation due to age or BMI was detected. The relative abundances globally shifted in favor of a pro-inflammatory profile that ultimately drove negative metabolic effects including decreased insulin sensitivity through the HOMA-IR index and oral glucose tolerance test (OGTT). This is exemplified through decreased insulin sensitivity at fasted and fed states in the sleep-deprived phase with respect to the recovery phase. The direction of change for the Tenericutes phylum following sleep deprivation contrasts with the findings of Liu et al. [34]., which warrants further research in this phylum.

Another study using a similar population as Wang et al. [13]. followed participants through two baseline days, after which they were subjected to a 40-h sleep deprivation cycle and one night of recovery. Gut composition analysis denoted a decreased relative abundance of numerous genuses, including *Prevotella* and *Parasuturella*. Additional analysis illustrated a reduction in gut total SCFA, namely acetate, propionate, and butyrate due to sleep deprivation. The study further only transplanted the sleep-deprived fecal samples from participants into germ-free mice resulting in systemic inflammation and most noticeably neuroinflammation. Overall, the study substantiates the detrimental consequences of sleep deprivation in humans and, to a larger extent, in mice.

Lastly, Reutrakul et al. [30]. followed eight healthy adults through two-weeks of home sleep extension and two weeks of habitual sleep. Actigraphy-based analyses did not reveal any significant intra- and inter-individual bacterial diversity shifts through sleep extension. Further analyses through the PSQI questionnaire showed a positive correlation between sleep efficiency with the Tenericutes phyla relative abundance.

## Discussion

The present review systematically appraised the gut microbiota composition associated with sleep quality and duration following induced sleep disturbances from six cross-sectional and five experimental clinical studies in healthy individuals aged 4–71. The six cross-sectional studies showed how poor sleep as measured by parameters including sleep efficiency, quality, and duration was associated with altered gut microbial composition and bacterial diversity. Interestingly, these studies also comparatively displayed similarities and differences depending on the age of the individuals studied in gut microbiome diversity and composition. The majority of clinical trials revealed the impact of sleep disturbance on gut microbial composition and diversity changes through short-term sleep-restricting protocols. Overall, sleep efficiency is an important factor that affects the composition and bacterial diversity of gut microbiota regardless of age (Figure 4).

An array of studies determined changes in gut microbial composition with particular clinical relevance. The two dominant phyla in the gut are Firmicutes and Bacteroidetes, which account for 90% of gut bacterial composition [35]. The Firmicutes to Bacteroidetes ratio (F/B ratio) is commonly used as a proxy for metabolic homeostasis [36, 37]. However, the relative abundances and proportions of bacterial genera and strains vary between individuals across the lifespan. In younger adults, superior sleep quality is associated with an increase in the F/B ratio [31]. Similarly, the gene sequencing analysis in young adults [15] also exhibited a positive correlation between sleep efficiency with both Firmicutes and Bacteroidetes phyla. Studies investigating sleep duration restriction addressed differences in the Firmicutes phyla in preschool aged children [32] and in young adults [27]. Bacteria under the Firmicutes phylum are generally associated with their butyrate-producing capacity and their ability to maintain a healthy gut [38]. Among the SCFAs, butyrate possesses substantial anti-inflammatory capacities by regulating an array of immune cells [39]. Moreover, preschool aged children [32] also notably revealed a decrease in *Bifidobacterium* genus in short duration sleepers, which is known for nutrient breakdown and absorption, acetate production, and promotion of gut barrier integrity [40, 41]. Likewise, young adults observed a negative correlation between bacterial species and sleep quality and the abundance of *Blautia*genus [15]. Likewise, Wang et al. [13]. The *Blautia* genus belongs to the Firmicutes phylum that is positively correlated with the pro-inflammatory cytokine IL-1β in chronically sleep-disturbed as opposed to healthy individuals [42]. These observational studies support an association between sleep parameters and bacterial composition, but there is no agreement on the compositional changes of specific bacteria.

Two cross-sectional studies specifically focused on older adults. One study particularly focused on sleep quality and its impact on taxa abundances [26]. The results reflect an increase in Verrucomicrobia and Lentisphaerae phyla. The increase in Verrucomicrobia may pose significant health benefits, namely through the *Akkermansia muciniphila* species, *A. muciniphila* is a designated gate-keeper of the gut epithelial layer and is known to promote gut barrier integrity, preventing pathogenic and pro-inflammatory bacteria from entering circulation [43]. However, the concerning increase in abundance of the opportunistic pathogen family *Pseudeomonas*in the study of Agrawal et al. [33] experimenting with healthy older adults may pose a serious risk. The consequences of high abundances in *Pseudeomonas*, specifically the *aeruginosa* species, has been documented in children. Overgrowth of *P. aeruginosa* enters the bloodstream via gut epithelial cells, thus promoting sepsis-induced generalized inflammation [44, 45]. In older adults, other chronic conditions including diabetes mellitus could further predispose sufferers to higher *P. aeruginosa* concentrations, perpetuating a muscle-catabolic systemic low-grade inflammation [46].

Findings from experimental clinical studies in young adults offered a mixed interpretation regarding the effects of sleep disturbance on gut microbiome composition. Firstly, both Zhang et al. [28]. and Liu et al. [34]. detected a higher F/B ratio due to sleep disruption, Furthermore, Wang et al. [13]. demonstrated a negative correlation between sleep duration and bacterial diversity, however, Reutrakul et al. [30]. did not observe similar findings. Aside from finding no bacterial diversity differences between short and long duration sleepers, low sleepers showed a decreased abundance in Tenericutes [30]. Another study [28] corroborated a decrease of Tenericutes in short vs normal sleepers, whereas Liu et al. [34]. highlighted an increased Tenericutes abundance. There is currently not enough evidence to assign either a commensal or pathogenic role to Tenericutes in host physiology. Zhang et al. [28]. highlighted an increase in Fusobacteria and Proteobacteria phyla in short sleepers vs normal sleepers, which are both linked with low-grade inflammation [47, 48]. This study also revealed that levels of Tenericutes decreased in short vs normal sleepers. The aforementioned clinical studies support a decreased relative abundance of commensal bacteria with a concomitant increase of potential pathogenic bacteria after sleep deprivation. Nevertheless, experimental studies exploring sleep disturbance in gut microbial composition of older populations are needed to understand the directionality of the relationship across the lifespan.

The findings of the present systematic review are consistent with those of the general literature. Changes in gut microbiome composition have been confirmed in studies investigating the effect of sleep disturbance in individuals with chronic comorbidities, including depression [49, 50] and Alzheimer’s disease [51]. The theoretical underpinning is that sleep and the gut microbiome have a cyclical relationship through immune, metabolic and neuroendocrine pathways [52]. On one hand, sleep disturbance and psychiatric conditions can lead to gut dysbiosis through neuroendocrine and immune responses. Specifically, sleep disturbance is proposed to be linked with activation of the cortisol-producing hypothalamic-pituitary-adrenal (HPA) axis, altering gut bacterial composition and reducing intestinal barrier function [53, 54]. Interestingly, the bacterial changes may stimulate pro-inflammatory cytokines including TNF-alpha and IL-6, that may further exacerbate sleep disturbance [55]. On the other hand, bacterial metabolites such as the SCFA butyrate can directly communicate via the vagus nerve and act as a signaling molecule to induce sleep onset [56]. The scenario changes radically in pathophysiological conditions such as insomnia, a prevalent sleeping disorder in older adults associated with pro-inflammatory markers IL-6 and C-reactive protein [57–59]. A recent study associated lower levels of sleep efficiency to higher levels of fecal SCFA in older adults. A proposed mechanism may be the low absorption of SCFA through gut epithelial cells into circulation, thus potentially perpetuating low-grade systemic inflammation [60]. However, the specific metabolic link between insomnia and inflammation is still poorly understood. Moreover, traditional sleep-related factors such as γ-aminobutyric acid (GABA) may also be produced from several gut bacterial species that are positively associated with sleep duration [61]. Therefore, reduced gut-derived GABA could inherently reduce sleep duration via the vagus nerve [62–64]. Overall, the gut-brain axis offers a valid explanation in understanding the effects of sleep disturbance on gut microbiome composition.

### A case for sarcopenia

The complex pathophysiology of sarcopenia is underlined, in part, by chronic low-grade systemic inflammation [65]. Main drivers of chronic systemic inflammation include chronic infections, obesity and major hallmarks of aging such as oxidative stress, immune dysregulation and cellular senescence [66, 67]. However, anabolic resistance also plays a major role in the development of sarcopenia through senescence-independent inflammation. In fact, the gut microbiome may contribute to anabolic resistance through an altered bacterial profile. For instance, a recent study associated sarcopenia severity in older adults to a high abundance of six bacterial species, with *Desulfovibriopiger*as as the primary driver [68]. This sulfate-reducing bacteria is speculated to contribute towards Inflammatory Bowel Disease (IBD) through the production of hydrogen sulfide, a cytotoxic compound to the gut epithelium [69]. An additional study in older adults denoted an altered gut microbiome composition, namely through an increase in mucin-degrading *Anaerotruncus* and a decrease in polysaccharide-digesting commensal *Prevotella* [70]. The increase in *Anaerotruncus* may contribute to the breakdown of gut barrier mucin, thus increasing gut permeability and promoting systematic inflammation [71]. Interestingly, the reduction in *Prevotella* may point towards an array of confounding factors that may additionally favor the onset of sarcopenia via the gut microbiome [72]. Modifiable risk factors such as nutrition, physical activity, and sleep may significantly contribute towards or hinder the onset of sarcopenia. Nutrition and physical activity are directly related to muscle health and may be mediated by the gut microbiome through nutrient absorption and SCFAs that regulate insulin sensitivity; a pivotal regulator of muscle growth [73, 74]. Conversely, disrupted sleep has been associated to muscle mass and strength losses, yet the mediating role of the gut microbiome remains to be established [75]. To evaluate the potential relationship between sleep disruption, gut microbiome, and muscle health, focus should be given to studies investigating such link in controlled conditions, such as shiftwork.

The nature of shift-work disrupts the regular light exposure, sleep quality and length, and dietary patterns. These in turn may lead to altered biological patterns that overall dysregulate muscle protein balance through hormonal imbalances and disrupted protein intake timings [76]. For these reasons, shiftwork has an established association with overweight or obesity and numerous chronic diseases [77–79]. Interestingly, a study in middle-aged shift workers denoted distinct biomarkers depending on day or night shiftwork [80]. Higher abundance of *Faecalbacterium* in day shiftworkers has been associated with healthier gut profiles due to its butyrate-producing anti-inflammatory capacity [81]. Contrarily, night shift work has been associated with an increase in *Dorea*, which has previously been correlated to promoting type II diabetes mellitus [82]. An additional study within the same cohort revealed that abundances of certain bacteria were strengthened or aggravated in night shift workers consuming certain diets [83]. For example, *Ruminococcusgnavus* was significantly higher in night shift workers and further abundant in those with a high-sugar diet. This species is notorious for producing lipopolysaccharides (LPS) that may contribute to the onset of Crohn’s disease [84]. Therefore, sleep deprivation is linked to a muscle-catabolic environment [11], yet the exact mediating role of the sleep-induced microbial changes in anabolic resistance remains unclear [85]. The present paper further proposes a molecular pathway by which gut health could lead to anabolic resistance as seen in sarcopenia.

**Figure 5.**
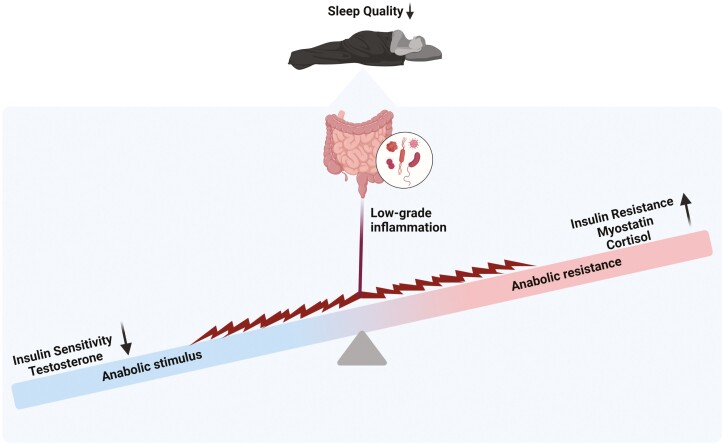
Proposed mechanism by which sleep disruption contributes to sarcopenia-related muscle dysfunction via the gut-muscle axis.

Poor sleep has been linked with an increased risk of skeletal muscle dysfunction, which may be partially explained by a shift of microbial species to pro-inflammatory bacteria [86]. In recent years, multiple articles have suggested a potential role of gut bacteria involved in the aetiology of sarcopenia, namely through the gut-muscle axis [87–90] (Figure 5). In particular, the modulatory role of pathogenic gut bacteria in disturbing physiological homeostasis and gut barrier integrity via the production of LPS has been proposed as a hallmark of anabolic resistance. Sleep disturbance can act as a trigger to promote low-grade systemic inflammation through LPS-induced pro-inflammatory cascades [91]. LPS enters the circulation through the permeable gut barrier in a dysbiotic gut [92, 93], triggering the systemic activation of pro-inflammatory cytokines through the Toll-like receptor 4 (TLR4) pathway [94]. The immune cascade stimulates systemic inflammation—a pathological process whereby the pro-inflammatory cytokines increasingly damage vital organs and tissues [95]. A consequence of the systemic inflammation is the TLR4-mediated decreased insulin sensitivity of skeletal muscle, resulting in the activation of ubiquitin-proteasome and autophagy-lysosomal muscle-catabolic pathways [93, 94, 96]. The consequent muscle mass loss is correlated with the loss of muscle function, exemplified through lessened handgrip strength [97, 98] and gait speed [99–101]. Evidence also suggests a concomitant reduction in muscle quality through the loss of motor units and contractile properties of the muscle [102–105]. Ultimately, poor sleep may contribute to sarcopenia-related muscle dysfunction via a gut-muscle axis.

### Strengths and limitations

The reviewed studies presented a series of strengths. Specifically, the moderately high methodological quality of the included studies reflects robust procedures in both the observational and clinical trials. All studies used the standard method of 16S rRNA sequencing to determine gut microbiome composition, which allows for comparability among studies in terms of bacterial diversity and relative bacterial abundance. Furthermore, the expanding interest of the gut microbiome field has promoted innovative techniques to analyze bacterial interrelationships such as cooccurrence networks as depicted by Liu et al. [34]. Nevertheless, the array of modifiable risk factors for gut dysbiosis inevitably complicate research to establish causal links between lifestyle factors and the gut microbiome. These complications produce several limitations in sleep research. In particular, the first limitation consists of the confounders presented in lifestyle-related studies. Changes in gut microbiome composition may not be directly attributed to sleep and may be perplexed by factors such as genetics, sex, dietary patterns, exercise levels, and antibiotic use. This concerns cross-sectional studies that do not collect related data, while clinical trials may attempt to control these such as through standardized meal and physical activity diaries. From the included studies, only one observational study administered a Food Frequency Questionnaire to account for the diet-gut microbiome interaction [26]. However, the results are not included in the preliminary report. Regarding the five experimental clinical studies, three asked participants to adhere to their regular meal timings [13, 29, 34]. One study provided ad libitum access to food during the sleep protocols [28]. The fifth study administered three-day food diaries during each sleep period, but the analysis did not account for the covariate [106]. Moreover, the length of clinical study protocols only allows to speculate the impact of sleep on gut bacteria in the short-term. While practically challenging, longer-term studies would potentially identify a series of gut biomarkers associated to sleep parameters. The heterogeneity in sleep measure parameters through subjective self-reported questionnaires and objective accelerometery make it difficult to standardize and estimate the size of impact of sleep on microbial composition. Promoting the use of well-established questionnaires such as PSQI, may synergistically help overcome their respective limitations [107]. Eventually, an additional limitation is directly related the design of cross-sectional studies, often part of larger cohort studies. Their results do not allow to speculate any sense directionality or temporality. Thus, promoting intervention studies could help establishing these between sleep disturbance and microbial composition.

## Conclusions

Our systematic review illustrates how sleep disturbance may affect sleep quality, duration, and efficiency, leading to shifts in gut microbiome composition and bacterial diversity. Specific similarities throughout the lifespan are addressed in terms of sleep quality with bacterial diversity and differences for sleep duration with taxa abundance. Pathological bacterial profiles may promote manifestations involved in the development of chronic low-grade inflammation that could be a factor in the development of age-related sarcopenia. The findings of this systematic review warrant further research by investigating the modulatory effect of the gut microbiome between sleep disturbance and muscle dysfunction during aging.

## Supplementary Material

zsac239_suppl_Supplementary_Table_S1

## Data Availability

The data that support the findings of this review are available from the corresponding author upon reasonable request.
