# Supplementary material for: Gut microbiome changes due to sleep disruption in older and younger individuals: a case for sarcopenia?
Source: Sleep. 2022 Oct 2;45(12):zsac239. doi: 10.1093/sleep/zsac239 (PMC9742900; doi:10.1093/sleep/zsac239)
Supplement: zsac239_suppl_Supplementary_Table_S1 [file zsac239_suppl_supplementary_table_s1.docx]

**Gut microbiome changes due to sleep disruption in older and younger individuals: a case for sarcopenia?**

Jordi Morwani-Mangnani^1^, Panagiotis Giannos^2^, Clara Belzer^3^, Marian Beekman^1^, P. Eline Slagboom^1^, Konstantinos Prokopidis^4^

**^1^**Section of Molecular Epidemiology, Department of Biomedical Data Sciences, Leiden University Medical Center, Leiden, Netherlands

^2^Department of Life Sciences, Faculty of Natural Sciences, Imperial College London, London, UK

^3^Laboratory of Microbiology, Wageningen University, Netherlands

^4^Department of Musculoskeletal Biology, Institute of Life Course and Medical Sciences, University of Liverpool, Liverpool, UK

Corresponding Author: Jordi Morwani-Mangnani

Email: [j.morwani_mangnani@lumc.nl](mailto:j.morwani_mangnani@lumc.nl)

ORCID-ID: 0000-0003-4654-9317

**Table S1.**Search terms employed in the screening based on title, abstract and keywords in the literature search.

| **Database** | **Search terms** |  |
| --- | --- | --- |
|  |  |  |
| PubMed | ((gut microbio* [Title/Abstract] OR microflora [Title/Abstract] OR intestinal flora [Title/Abstract] OR gut dysbiosis) AND (sleep [Title/Abstract] OR sleep disorder [Title/Abstract])) |  |
| Cochrane Library | ((gut microbio* [Title/Abstract] OR microflora [Title/Abstract] OR intestinal flora [Title/Abstract] AND (sleep [Title/Abstract] OR sleep disorder [Title/Abstract] OR sleep fragmentation [Title/Abstract)) |  |
| Scopus | (((gut OR microbio*) AND (intestinal AND flora) AND (gut AND dysbiosis) AND (sleep OR disorder) OR (sleep AND deprivation))) |  |
| Web of Science | (((gut microbio* OR intestinal flora OR gut dysbiosis) AND (sleep disorder OR sleep deprivation OR sleep fragmentation OR sleep))) |  |
